# Papillomavirus Infection as Potential Cause of Miscarriage in the Early Gestational Age: A Prospective Study

**DOI:** 10.3390/diagnostics13091659

**Published:** 2023-05-08

**Authors:** Maria Teresa Bruno, Salvatore Caruso, Guido Scalia, Maria Costanzo, Salvatore Di Pasqua, Sara Boemi, Marco Marzio Panella, Marco Palumbo

**Affiliations:** 1 Department of General Surgery and Medical Surgery Specialties, Gynecological Clinic, University of Catania, 95123 Catania, Italy; 2 Multidisciplinary Research Center in Papillomavirus Pathology, University of Catania, 95123 Catania, Italy; 3 Department of Biomedical and Biotechnological Sciences, Clinical Virology, University of Catania, 95123 Catania, Italy

**Keywords:** miscarriage, gestational age, HPV infection, chorionic villi, adverse effects of pregnancy

## Abstract

The possible association between human papillomavirus (HPV) infection and negative pregnancy outcomes has been debated in the literature, with conflicting results from clinical trials. While some authors support a link between HPV and miscarriage, others argue that the mere detection of the virus does not necessarily indicate a causal relationship with negative pregnancy outcomes. In this study, we conducted a prospective, controlled investigation of the potential association between HPV infection and miscarriage. Our study included 59 women who had experienced a miscarriage and 57 women who had undergone voluntary termination of pregnancy (TOP) within the 12th week of gestation. We assessed HPV prevalence, maternal age, and HPV genotype in both groups and evaluated the relationship between these factors and pregnancy outcome. Unlike previous studies that only identified HPV in cases of abortion, we also correlated the positivity of chorionic villi with gestational age in both groups. We found a close correlation between positive chorionic villi and very early gestational age, with all 13 cases of virus-positive chorionic villi in the miscarriage group occurring in gestational periods of less than 8 + 5 weeks (<60 days) (RR = 28.6). Our analysis showed no correlation between HPV infection and maternal age or viral genotypes. The results suggest that the presence of HPV alone is not enough to cause spontaneous abortion, but a high viral load in early pregnancy may increase the risk of negative outcomes. These findings have important implications for the management of HPV infection during pregnancy and may provide a rationale for the use of HPV vaccines to reduce the incidence of spontaneous abortion and infertility due to preclinical spontaneous abortions.

## 1. Introduction

HPV infection is the most common sexually transmitted infection worldwide [1]. Approximately 12 types of HPV are considered high risk for cervical cancer. Two of these types (HPV 16 and HPV 18) cause approximately 7 out of 10 (70%) cervical cancer cases [2,3]. The virus has a strong affinity for the squamous epithelium of the uterine cervix, and although most cases resolve spontaneously within 1–2 years, persistent HPV infection can lead to cervical cancer [4]. A meta-analysis reported an overall prevalence of HPV in pregnant and non-pregnant women of the same age of 16.8% and 12.3%, respectively [5]. Pregnant women are more susceptible to HPV infection, and as gestational age increases, so does the prevalence of HPV during pregnancy, with higher rates seen in the second and third trimesters compared to the first trimester [6,7,8].

The increased incidence of HPV infection in pregnant women and the recent evidence that papillomavirus is capable of infecting and replicating in the trophoblast [9] have raised the suspicion that papillomavirus, like other viruses, can also adversely affect the outcome of pregnancy [10,11,12,13], given that the trophoblast plays a crucial role in the placentation process. In vitro evidence that HPV16 is able to complete its life cycle in trophoblastic cells has been reported by Liu et al. [9]. You et al. [14] found the active expression of the viral genome (both early and late genes) in trophoblastic 3A cells previously cultured with HPV 16, 18, 11, and 31. They also found that HPV31 infection and HPV16 E6 and E7 oncogenes caused both a decrease in the number of 3A trophoblastic cells and low adhesion between trophoblasts and endometrial cells in the first week after exposure in several in vitro studies [14,15]. These effects were also confirmed by Gomez et al., who found a three to six times higher rate of apoptosis in trophoblastic cells transfected with a plasmid containing the entire HPV16 genome and a progressive decrease in trophoblast invasion capacity from day 3 to day 15 after transfection compared to negative controls [10]. Hong et al. also showed a similar trend, with a reduction in both implantation rate (less than 37.2%) and migratory/invasive activity in embryos exposed to HPV16 [16].

Therefore, although in vitro studies [14,17] and animal models [16,18,19] have demonstrated the biological plausibility of the harmful effects of HPV on pregnancy outcomes, due to bias and the small number of published studies, the clinical evidence has produced conflicting conclusions. HPV DNA was detected with a wide prevalence range of 4–75% in the placenta of women who had had abortions and in 20–24% of women who had undergone a voluntary termination of pregnancy [12]. In contrast, Eppel et al. [20] found no HPV DNA in any of the 147 placental villus samples collected by trans-abdominal amniocentesis. Since the most frequent adverse effect of pregnancy is miscarriage, we conducted a prospective, controlled study to investigate the possible association between HPV infection and miscarriage.

## 2. Materials and Methods

### 2.1. Population

In this study, we used a prospective, case-control design to investigate the relationship between HPV infection and pregnancy loss. The study group consisted of women who had experienced a miscarriage (SA) within the 12th week, while the control group consisted of women who had undergone voluntary termination of pregnancy (TOP) within the 12th week. Between May 2020 and June 2021, we collected and analyzed chorionic villi samples from the placentas of 59 women in the study group and 57 women in the control group. We used HPV genotype detection to assess the prevalence and concordance of HPV infection in both the mother’s cervix and the chorionic villi of the placenta in the two groups. Additionally, we examined the relationship between positive chorionic villus and gestational age. The gestational age of the fetus was calculated with an ultrasound dating that detected the week/day that fetal growth had stopped. In the SA group, the gestational age was between the 6th and 12th week. In the TOP group, the gestational age was between the 7th and 12th week, and 82% of the women had a gestational age > the 10th week. We also correlated the gestational age with positive chorionic villus.

The sample size was calculated using EpiInfo, (7.2.0.1., Atlanta, GA, USA) with a 1:1 case-to-control ratio (95% confidence, 90% potency), for a total of 50 cases and 50 controls. The sample size was calculated by predicting a 10% difference in terms of HPV prevalence in patients of the SA group compared to the TOP group. A total of 67 patients would be required for the study endpoint using an alpha of 0.0500 and a potency of 90%, taking into account an HPV prevalence in the TOP group of 15.2%. The sample size was increased by 20%, requiring 80 subjects in order to correct for possible data loss.

Inclusion criteria: we enrolled pregnant women aged 18–40 who wanted termination of pregnancy (TOP) within the 12th week, as well as pregnant women who had had a miscarriage (within the 12th week) with negative Torch agents.

Exclusion criteria: women vaccinated against HPV, women with chromosomal abnormalities, autoimmune or genetic diseases such as LUPUS or thrombophilia, pregnant women with positive IgM Torch, women with any cause that may have justified a miscarriage such as uterine myoma, uterine malformation, and cervical-segmental incompetence.

The prospective study was approved by the Catania 1 Ethics Committee (No. 154/2019/OP) of the University Hospital of Catania, Italy, and informed written consent of all study participants was obtained. Participants responded to a questionnaire with demographic and clinical data in order to characterize the study group (Supplementary File).

### 2.2. Chorionic Villus Sampling Technique

To avoid contamination of the ovular material with HPV when crossing the maternal vagina, a special method was used. We induced dilatation of the cervix with the use of an oral tablet of mifepristone (200 mg). We inserted a 10 mm Karman cannula (İzmir/Turkey) into the already dilated cervix and connected it to an aspirator. This cannula acted as a sheath, through which a 6 mm Karman cannula reached the uterine cavity and aspirated the ovular material without contaminating it. We studied the fetal part of the placenta; that is, the chorionic villi that were carefully characterized by the pathologist as fetal (chorionic villi) or maternal (deciduous) in origin.

We chose to work on fresh, non-paraffin embedded placental tissue in order to make the samples as homogeneous as possible. The placental tissue was processed under a sterile hood, and, using a petri dish as a support, was reduced to very small pieces using a scalpel from which 3 small sections were taken from 3 different points and placed in an Eppendorf (Milan, Italy) type tube. Subsequently, treatment continued with cell lysis by adding lysis buffer (ATL swab) (Milan, Italy) and proteinase K (Madison, WI, USA) with an overnight incubation at 55 °C. The next day, extraction continued using the high, pure PCR Template (ROCHE) commercial kit (Roche Diagnostic GmbH, Mannheim, Germany) that is based on the ability of DNA to bind to inert media or filters contained in the columns. Subsequently, the nucleic acids bound to the filter were eluted by elution buffer and the eluate was stored at −80 °C until the time of use. Details of the technique are described in the reference [21].

### 2.3. HPV Test and Genotyping

After cytological sampling for HPV DNA, samples were sent to the laboratory for DNA extraction and viral DNA genotyping by genetic amplification followed by hybridization with genotype-specific probes capable of identifying most of the HPV genotypes of the genital region [18 high-risk HPV genotypes (16, 18, 26, 31, 33, 35, 39, 45, 51, 52, 53, 56, 58, 59, 66, 68, 73, 82), 7 low risk (6, 11, 40, 43, 44, 54, 70) and 3 undefined risk (69, 71, 74)]. The commercial method used was the MAG NucliSenseasy system (bioMérieux SA, Marcy l’Etoile, France). The technique was previously described [14]. The DNA amplification technique was previously described in reference [22].

### 2.4. Statistical Analysis

The statistical analysis of the data was carried out using the SPSS 15.0 software package (SPSS Inc.; Chicago, IL, USA). Descriptive statistics were expressed as frequency, arithmetic mean, and percentages. The results are summarized in Table 1, Table 2, Table 3, Table 4, Table 5 and Table 6. The relationship between the categorical variables was evaluated by Chi-square tests. The correlation between age, HPV prevalence, and gestational age were evaluated by estimating Relative Risk (RR) with confidence intervals (CI) of 95%. Statistical significance was defined as *p* < 0.05.

## 3. Results

Seven women from the SA group and four from the TOP group were excluded from the study due to insufficient placental material. Two women from the study group and three women from the control group were excluded because they did not undergo HPV testing of the cervix. Therefore, the SA group consisted of 50 women with spontaneous abortion within the 12th week, and the TOP group consisted of 50 women who voluntarily aborted within the 12th week. 

Our findings showed that 34.0% (17/50) of women in each group had an HPV positive cervix. There were 22 cases of positive chorionic villi, with 13 cases (26.0%) in the SA group and nine cases (18.0%) in the TOP group. Of the 34 women with an HPV positive cervix, only 15 (44.1%) also had positive chorionic villi, while 19 (55.9%) had negative chorionic villi. Additionally, seven women with an HPV negative cervix had positive chorionic villi. 

In the SA group, we found 34.0% (17/50) of women with an HPV positive cervix and 26.0% (13/50) with positive chorionic villi. Of these, nine cases had the same corresponding HPV genotype in the uterine cervix, while four cases belonged to women with an HPV negative cervix. Furthermore, 33 women with an HPV negative cervix had four cases of positive chorionic villi and 29 cases of negative chorionic villi (Table 1).

**Table 1:** Distribution of HPV infection in the cervix and chorionic villi in the study group and control group.

Study Group (SA)			Control Group (TOP)		
Cervix	Chorionic Villi		Cervix	Chorionic Villi	
	Positive	Negative		Positive	Negative
Positive 17 (34%)	9	8	Positive 17 (34%)	6	11
Negative 33 (66%)	4	29	Negative 33 (66%)	3	30

SA: Spontaneous abortion or miscarriage; TOP: women who had resorted to voluntary termination of pregnancy.

In the SA group, the estimated relative risk (RR) was found to be 4.4 (95% CI: 1.6, 12.1), *p* = 0.0018, indicating that the risk of having positive chorionic villi when the cervix is positive was four times higher compared to when the cervix is negative. This difference was statistically significant.

In the control group, 34.0% (17/50) of women had an HPV positive cervix, and 18.0% (9/50) had positive chorionic villi. Of these, six cases had the corresponding genotype in the uterine cervix, and three cases belonged to the women with HPV negative cervixes. Additionally, 33 women had an HPV negative cervix, of which three cases were positive for villi and 30 cases were negative for villi (Table 2).

**Table 2:** The risk of having positive chorionic villi in both groups.

Study Group			Control Group		
RR	95% CI	*p* Value	RR	95% CI	*p* Value
**4.4**	16–12.1	0.0018	3.9	1.1–1.6	0.0223

The relative risk in the control group was estimated to be 3.9 (95% CI: 1.1–13.6) with a *p*-value of 0.0223. The differences between the two groups were not statistically significant, RR = 1.50 (95% CI = 0.68–3.3), *p* = 0.300.

The concordance of genotypes between positive cervix and positive chorionic villi occurred in 100% of miscarriage cases. In the control group, in two cases of multiple infection (51–53 and 16–31), only one of the genotypes was found in chorionic villi (HPV 53 and HPV 16), and one woman whose cervix was positive for a genotype (HPV 67) the villi were positive for a different genotype (HPV 59).

In the maternal cervixes of the SA group, the most frequent genotype was HPV16 (five cases), followed by 31 (three cases), 61 (two cases), then 51, 90, 87, 59, 40, 67, 73, 53, 58, 53, 81, 82, 42, and 54. The nine cases of positive chorionic villi had genotypes corresponding to those of the cervix: HPV 16 (4 cases), and 51, 61, 67, 54, 31, 53, 73, and 42. In four cases, the chorionic villi were positive for the following genotypes: 61, 31, 51, and 6 with the maternal cervix being HPV negative (Table 3).

**Table 3:** Distribution of HPV genotypes in the cervix and chorionic villi of both groups.

***n***	**Cervix**	**Genotypes**	**Chorionic Villi**
**SA group**			
9	positive	51, 16, 61, 54, 16, 16, 16–42, 67–73, 31–53	positive
8	positive	61, 16–18, 31–42–53, 90, 87, 59–62, 40, 31–54–73–81–82	negative
4	negative	31, 61, 6, 51	positive
29	negative	negative	negative
**TOP group**			
6	positive	6–56, 90, 51–53, 16–31, 6, 67	positive
11	positive	16, 16, 31, 31, 67, 66, 62, 52, 66, 31–81	negative
3	negative	16–52, 35, 54	positive
30	negative	negative	negative

In the control group, regarding the maternal cervix, the most frequent genotype was HPV31 (four cases), followed by HPV 16 (three cases), 66, 62, 51, and 6 (two cases), and genotypes 90, 81, 67, 56, 53, 52, and 39. The chorionic villi had only one case of HPV 16 corresponding to the cervix, no cases of HPV 31, and there was correspondence between the cervix and villi with 6 and 90 genotypes, respectively. One woman had a positive cervix for the 67 genotype and positive villi for the 59 genotype. In addition, there were three positive cases for genotypes 54, 35, and 16–52 with HPV negative cervixes. The difference between the SA and TOP groups was not statistically significant (*p=* 0.23).

We also stratified the two groups according to gestational age. The SA group had a gestational age between sixth and 12th weeks, and the women who had resorted to abortion had a gestational age between the seventh and 12th week (Table 4).

**Table 4:** Gestational age and positive chorionic villi in women with miscarriage and TOP.

Gestational Age ≤8 Weeks vs. >8 Weeks (Cut-Off)					
Study Group (SA)			Control Group (TOP)		
RR	95% CI	*p* Value	RR	95% CI	*p* Value
**28.6**	4.1–200.5	5.17 × 10^−9^	0.25	0.016–3.9	0.252

In the SA group, the vast majority of women with a gestational age between the sixth and 8th weeks exhibited positive chorionic villi (81.3%, 13/16), while only a small proportion had negative villi (18.8%, 3/16). In contrast, women with a gestational age greater than 8 + 4 weeks exhibited exclusively negative chorionic villi. By using 8 + 4 weeks as the cut-off, the maximum difference in the estimated RR was observed, with an RR = 28.6 (95% CI: 4.1–200.5, *p* < 0.001) (Table 5).

**Table 5:** The maximum difference between the relative risk (RR) in the two groups was obtained by considering the cut-off at 8 + 4 weeks.

Study Group: SA						Control Group: TOP					
Gestational Age		Cervix		Chorionic Villi		Gestational Age		Cervix		Chorionic Villi	
Weeks	*n*	Positive	Negative	Positive	Negative	Weeks	*n*	Positive	Negative	Positive	Negative
6–8	16	9	7	13	3	**7–8**	8	2	6	0	8
9–10	22	3	19	0	22	**9–10**	17	8	9	4	13
11–12	12	5	7	0	12	**11–12**	25	7	18	5	20

In the study group, the age group of ≤30 years comprised 17 women (mean age 25.2). Of these, six women had an HPV-positive cervix, including four cases with genotypes corresponding to the chorionic villi genotypes, two cases with negative villi, two cases with positive villi and HPV-negative cervixes, and nine cases with negative results in both cervix and placenta. The age group of >30 years comprised 33 women (mean age 36.5). Among them, 11 women had an HPV-positive cervix, with five cases having genotypes corresponding to the chorionic villi genotypes and six cases with negative villi. We found two cases of positive chorionic villi in women with an HPV-negative cervix. Twenty women with HPV-negative cervixes had negative villi (Table 6).

**Table 6:** Study group and control group divided by age.

Study Group (SA)						Control Group (TOP)					
<30 Anni (17 Cases)				>30 Anni (33 Cases)		<30 Anni (28 Cases)			>30 Anni (22 Cases)		
*n* Cases	Cervix HPV Positive	Chorionic Villi HPV Poitive	*n* Cases	Cervix HPV Positive	Chorionic Villi HPV Positive	*n* Cases	Cervix HPV Positive	Chorionic Villi HPV Positive	*n* Cases	Cervix HPV Positive	Chorionic Villi HPV Positive
**4**	4	4	5	5	5	4	4	4	2	2	2
**2**	2	0	6	6	0	9	9	0	2	2	0
**2**	0	2	2	0	2	2	0	2	1	0	1
**9**	0	0	20	0	0	13	0	0	17	0	0

Women aged ≤30 years showed a slightly higher proportion of positive chorionic villi than women over 30 (RR = 1.5 95% CI: 0.6–3.5, *p* = 0.402), although this difference was not statistically significant. 

In the control group, the age group of women ≤ 30 years consisted of 28 individuals with a mean age of 25.5 years. Thirteen of them had an HPV positive cervix, including four with genotypes matching the ones found in the chorionic villi. Nine women had negative placental villi. Two cases of HPV-negative cervixes had positive villi. Thirteen women had negative results in both the cervix and placenta. The age group of women > 30 years consisted of 22 individuals with a mean age of 37 years. Among them, only four women had an HPV positive cervix, with two corresponding to genotypes found in the chorionic villi and two with negative villi. Two HPV negative cervix cases had positive HPV villi. Women over 30 had a slightly higher proportion of positive chorionic villi compared to women under 30, but the difference was not statistically significant, RR = 0.6 (95% CI: 0.2–2.2, *p* = 0.481).

After stratifying the female participants by age, we found that those aged ≤30 in the SA group had a lower incidence of cervical HPV compared to those in the TOP group of the same age. However, they had a higher incidence of positive chorionic villi. The RR for positive chorionic villi was 3.6 (95% CI: 0.9–14.5, *p* = 0.05) compared to those in the TOP group of the same age. The RR for cervical HPV was 2.3 (95% CI: 0.5–10.6, *p* = 0.26), although the difference was not statistically significant, with an RR of 2.2 (95% CI: 0.8–5.9, *p* = 0.14). 

Furthermore, women aged >30 in the SA group had a higher incidence of HPV at both the cervical (33.3%) and chorionic villi level (35.2%), with an RR of 5.0 (95% CI: 1.1–21.8, *p* = 0.02), than those in the TOP group of the same age (18.1% and 13.6%, respectively). The RR for cervical HPV was 9.0 (95% CI: 1.1–76.7, *p* = 0.02). However, the difference was not statistically significant, with a *p*-value of 0.88.

## 4. Discussion

To date, clinical studies have been able to demonstrate only the presence of papillomavirus in pregnant women [23,24] (Table S1). The results of our study suggest that the presence of HPV alone is not sufficient to cause a miscarriage, but a high viral load in early pregnancy may increase the risk of negative outcomes. These findings have important implications for the management of HPV infection during pregnancy and may provide a rationale for the use of HPV vaccines to reduce the incidence of miscarriage and infertility due to preclinical miscarriages. Studies focusing on the link between HPV and abortion are few and inconclusive due to biases related to different study methods, patient selection, different HPV detection methods used, maternal or gestational age, and the use of confounding variables. Many studies have focused only on cervical HPV detection [25,26,27], and very few have focused on placental detection of HPV [10,13,21,28,29] in pregnancy. Others have focused on searching for genotypes 16, 18, and a few other low-risk genotypes [10,13,24,29]. Some authors have used PCR to identify the virus, while others have used quantitative methods such as qPCR to detect DNA viruses [30]. Skoczynski et al. [28] found HPV DNA in 17.7% of miscarriage samples and 24.4% of placentas from full-term births. The choice of full-term births as a control group certainly negatively affected the association between HPV and miscarriage [25,28], given that a pregnant woman at term has a higher prevalence of HPV than in the first trimester of pregnancy (abortion). These biases make a reliable conclusion difficult. In our study, in order to avoid confounding variables, we compared AS women with TOP women of the same age and whose samples were collected within the 12th week of gestation. A careful selection was made to recruit women with unexplained miscarriages, and we excluded women positive for TORCH agents, women positive for HIV, HBV, HCV, and syphilis infections or on immunosuppressive therapy, as well as women with known causes of AS, such as uterine malformations, hormonal alterations, and chromosomal and genetic pathologies. In addition, to avoid contamination of the villi during vagina crossing, we used a procedure [21] that allowed us to aspirate ovulary material through a cannula (which acts as a sheath) that was introduced through the already-dilated cervical canal with the use of drugs. Considering that the cervix represents not only an anatomical barrier but also an immunological one against ascending infection, this method allowed us to take the chorionic villi from the intrauterine cavity without coming into contact with the vagina, which would probably have been infected.

We studied the fetal part of the placenta; that is, the chorionic villi that were identified by the pathologist as fetal (chorionic villi) or maternal (deciduous) in origin. We found 26% positive chorionic villi in women in the AS group. In agreement with the studies of Gomez [10] and Weyn [31], our study undoubtedly demonstrated the presence of HPV in chorionic villi, which is in line with studies reporting a higher prevalence of HPV in the placenta of abortions (26%) compared to the placenta of voluntarily interrupted pregnancies (18%) [13]. In the SA group, 52.9% of cases had a correspondence between positive maternal cervix and positive chorionic villi, but we found four cases of positive villi with a negative maternal cervix. This shows that the pathway leading to villi positivity is not only ascending or only from the cervix but may have also have paternal origins given the possible positivity of seminal fluid to HPV. This suggests a possible role of spermatozoa as vectors for HPV transfer to the fetal–placental unit [32,33]. Our study shows that papillomavirus DNA can infect the placenta, and that infection can occur as early as the first trimester of pregnancy. Both maternal age and genotyping were not statistically significant. To date, clinical studies have demonstrated only the presence of the virus in pregnant women. A systematic review by Ambühl reported a higher incidence (24.9%) of HPV among women with positive HPV miscarriages, but without giving a decisive role to HPV infection [12]. Certainly, a positive HPV villus is an indication of the susceptibility of trophoblast cells to viral infection in vivo. Therefore, chorionic villus involvement should be evidence of the ability of the papilloma virus to cause a miscarriage. This concept was reinforced when we studied the relationship between HPV-positive chorionic villi and gestational age. The strength of the present study is that, contrary to all previous studies that have shown only the presence of HPV in cases of abortion, we have correlated positive chorionic villi with gestational age.

Our results show a close correlation between chorionic villus positivity and very early gestational age. All 13 cases of chorionic villi positive for the virus had a miscarriage in an early gestational period of less than 8 + 5 weeks (<60 days) (RR = 28.6). Considering gestational age, the maximum difference between RRs occurred within the 8-week cut-off. Specifically, in the AS group, women with gestational ages between the sixth and eighth weeks had a very significant relative risk of 28.6 (95% CI: 4.1, 200.5) compared to women with gestational ages between the ninth and twelfth weeks. For the control group, the estimated relative risk was not significantly different from the value of 1; thus, the management age variable did not seem to be influential. How can we interpret this result? A recent study by Mosbah A et al. showed that the gestational age of preterm births was positively correlated with fetal viral load and matrix metalloproteinase 2 (MMP-2) gene expression rate, indicating that the time of the adverse event (preterm birth, abortion) was related to the HPV-borne virus [34]. Results from in vitro studies strongly suggest that most of the negative effects of HPV resulting in abortion occur during a very early stage of pregnancy [18]. All these data lead us to think that the mere presence of the virus is not enough to cause abortion without a high viral load at an early gestational age. The virus, which can be transported to the fetal placenta even by infected spermatozoa [32,33,35], can induce cellular alterations affecting the trophoblast, if it has a high viral load in the first weeks of gestation. In vitro experiments have detected these alterations, which can lead to a spontaneous abortion in the first weeks of gestation (preclinical abortion). This makes clinical research inadequate, as it cannot study cases of miscarriage which are not yet clinically detectable (<5 weeks). Higher viral loads can induce earlier miscarriages. The cases detected in our study could represent late abortions (> 5 weeks <8 + 5 weeks) caused by a lower viral load.

Depending on the viral load, we found that the following alterations of the trophoblast were responsible for adverse events: very early events (early abortion) in cases of high viral load, and late events (preterm birth, gestosis) in cases of a lower viral load. Of course, the clinical result, gestational age, and viral load are also linked to the immune status of a woman and the responsible genotype. The presence of placental villi with low viral load can determine a physiological pregnancy and corresponds to the clinical cases of isolation of the virus in the placenta (positive HPV villi) of physiological pregnancies. Matovina et al. studied the karyotype of positive chorionic villi (five cases) in women with miscarriages. Three samples had an abnormal karyotype and two samples had an undetermined karyotype [36]. This suggests the usefulness of undertaking a study on the association between trophoblastic HPV infection and the development of genetic abnormalities during embryogenesis. These results lead us to promote prospective and more numerous clinical studies aimed at studying the viral load (qPCR) of papillomavirus within the trophoblast in relation to gestational age. Tognon et al. [30] have recently investigated the association between HPV infection and miscarriage using both qualitative PCR and quantitative PCR. Unfortunately, they reported no reference to gestational age. It would be interesting to define the cytogenetics of HPV-positive villi cells in all those cases of unexplained abortions that occur in a very early gestational period (<60 days). The search for E6/E7 mRNA HPV would be useful, as this technique is widely used in the triage of HPV-positive women to increase the specificity of the HPVDNA test and would indicate the active viruses. However, its use in this context has yet to be well defined.

If our data are confirmed, the HPV vaccine not only improves the sex life of women with an HPV positive cervix [37], but also represents a fundamental means to reduce the incidence of spontaneous abortions and cases of couple infertility due to spontaneous preclinical abortions. The strengths of our study are that we considered a homogeneous series such as maternal age and gestational age in both groups, we carefully selected women with unexplained miscarriages, we used a technique that allowed us to exclude contamination through the vagina, and we used PCR genotyping to identify all possible genotypes. We looked for the virus in the chorionic villi (fetal part of the placenta) and in the cervix as many other studies have done. To date, ours is the only study that correlated the positivity of chorionic villi with gestational age, thus highlighting the correspondence of positive villi with early abortion (<60 days). The weaknesses of our study are the small sample size, the lack of use of a quantitative PCR technique that measures the viral load, and the lack of a cytogenetic study of HPV-positive villi cells. It could also be useful to search for HPV E6/E7mRNA, a marker used to increase the specificity of the HPV DNA test in HPV-positive women [38], which could identify the active infections among the genotypes detected in the placenta.

## 5. Conclusions

If our data are confirmed, the HPV vaccine not only improves the sex life of women with an HPV positive cervix, but represents a fundamental weapon to reduce the incidence of spontaneous abortions and all those cases of couple infertility due to spontaneous preclinical abortions.

## Data Availability

Data are contained within the article and the Supplementary Materials.

## Supplementary Materials

The following supporting information can be downloaded at: https://www.mdpi.com/article/10.3390/diagnostics13091659/s1, Table S1: Studies addressing the impact of HPV infection on pregnancy outcome.

## Abbreviations

SA	Spontaneous abortion or miscarriage
TOP	Terminator of pregnancy
HPV	Human Papilloma Virus
RR	Relative risk
PCR	Polymerase chain reaction
qPCR	Quantitative PCR
