# Supplementary material for: Papillomavirus Infection as Potential Cause of Miscarriage in the Early Gestational Age: A Prospective Study
Source: Diagnostics (Basel). 2023 May 8;13(9):1659. doi: 10.3390/diagnostics13091659 (PMC10178196; doi:10.3390/diagnostics13091659)
Supplement: Supplementary file 1 [file diagnostics-13-01659-s001.zip › diagnostics-2341718-supplementary.pdf]

Table S1. Studies addressing the impact of HPV infection on pregnancy outcome

Authors, years	Study Type	HPV Detection	HPV Type	Conclusions
**[21]**	Prospective Study (64 cases)	Chorionic villi	HPVDNA test, PCR	Positive villi
**[23]**	prospective study (899 cases)	placental tissue	HPV DNA test e genotyping (PCR)	preterm birth
**[30]**	Case-control study (80 cases miscarriage 80 cases TOP)	Chorionicvilli	PCR, qPCR	negative
**[11]**	Prospective Study (81 cases)	Decidual and Chorionic villi	HPV DNA, genotying	Miscarriage
**[32]**	case-control study	Chorionic villi	HPV DNA test, genotyping, qPCR	preterm delivery
**[28]**	Prospective Study case-control	Placental tissue	HPV DNA test, PCR	Miscarriage
**[12]**	Systematic literature search (42 studies)	Cervical samples placental tissue	HPV DNA test (PCR) Pathological examination of the placenta	Miscarriage
**[24]**	Case-control study (127 cases)	Cervical samples	HPV DNA test (PCR) Genotyping	negative
**[25]**	retrospective case-control study, (49 cases)	Cervical samples	HPV DNAtest, genotyping PCR	negative
**[31]**	Prospective study (35 cases)	Abdominal chorionic villi	HPV DNA test genotyping	positive villi
**[27]**	Prospective case-control study (51 cases with miscariage) women after term delivery (78)	placental tissue	HPV DNA test, PCR 16/18	negative
**[10]**	Case control study (108 cases)	Chorionic villi	HPV DNAtest genotyping 16, 18, 6, 11	preterm delivery
**[20]**	Prospective study (147 cases)	Abdominal chorionic villi	HPV DNA test, PCR	No HPV
**[13]**	Prospective study	Cervical samples	HPV DNA test, PCR	Miscarriage
